# The DNA of coral reef biodiversity: predicting and protecting genetic diversity of reef assemblages

**DOI:** 10.1098/rspb.2016.0354

**Published:** 2016-04-27

**Authors:** Kimberly A. Selkoe, Oscar E. Gaggiotti, Eric A. Treml, Johanna L. K. Wren, Mary K. Donovan, Hawai‘i Reef Connectivity Consortium, Robert J. Toonen

**Affiliations:** 1 Hawai‘i Institute of Marine Biology, University of Hawai‘i, Kāne‘ohe, HI 97644, USA; 2 National Center for Ecological Analysis and Synthesis, 735 State Street, Santa Barbara, CA 93101, USA; 3 School of Biology, Scottish Oceans Institute, University of St Andrews, St Andrews, Fife KY16 8LB, UK; 4 School of BioSciences, University of Melbourne, Parkville, Victoria 3010, Australia; 5 Department of Oceanography, University of Hawai‘i at Mānoa, Honolulu, HI 96822, USA; 6 Department of Biology, University of Hawai‘i at Mānoa, Honolulu, HI 96822, USA

**Keywords:** population genetics, seascape genetics, landscape genetics, resilience, coral reefs, ecosystem-based management

## Abstract

Conservation of ecological communities requires deepening our understanding of genetic diversity patterns and drivers at community-wide scales. Here, we use seascape genetic analysis of a diversity metric, allelic richness (AR), for 47 reef species sampled across 13 Hawaiian Islands to empirically demonstrate that large reefs high in coral cover harbour the greatest genetic diversity on average. We found that a species's life history (e.g. depth range and herbivory) mediates response of genetic diversity to seascape drivers in logical ways. Furthermore, a metric of combined multi-species AR showed strong coupling to species richness and habitat area, quality and stability that few species showed individually. We hypothesize that macro-ecological forces and species interactions, by mediating species turnover and occupancy (and thus a site's mean effective population size), influence the aggregate genetic diversity of a site, potentially allowing it to behave as an apparent emergent trait that is shaped by the dominant seascape drivers. The results highlight inherent feedbacks between ecology and genetics, raise concern that genetic resilience of entire reef communities is compromised by factors that reduce coral cover or available habitat, including thermal stress, and provide a foundation for new strategies for monitoring and preserving biodiversity of entire reef ecosystems.

## 1. Introduction

Known for their stunning arrays of colours, shapes and life forms, coral reefs are captivating examples of extreme biodiversity. Hidden within the taxonomic and life-history diversity found on reefs, but no less important, is the genetic diversity carried within individuals and populations. Genetic diversity is the seed of ecological and evolutionary processes like niche partitioning and species diversification that lead to the complex community structure typical of coral reefs and other highly biodiverse ecosystems. In turn, community-level processes no doubt have consequences for genetic diversity within populations, although mechanisms are not well studied. Because of the many possible ways in which genetic diversity may be linked to ecological functioning, adaptive capacity and extinction risk, conservation strategies often call for preserving areas of high genetic diversity [1,2]. When conservation strategies focus on habitats, communities or ecosystems, as is common for coral reefs, it is important to understand how genetic diversity patterns vary across co-distributed species, because including genetic data can shift conservation priorities dramatically [3].

Investigating patterns of genetic diversity at the community level can be framed by tests of foundational theory on drivers of biodiversity. Theory predicts that physical area constrains diversity by limiting carrying capacity and the genetic ‘effective’ population size, while immigration boosts diversity by bringing in new variants [4]. Species with similar constraints on habitat and movement may thus be expected to have similar patterns of genetic diversity [5], despite important trait differences across species [6,7]. Furthermore, historical events, such as major disturbances, may act on whole communities to produce a common signature of genetic bottleneck that depresses observed genetic diversity [7,8]. Accumulating evidence indicates that ecological interactions can shape genetic diversity, such as when the genetic diversity of habitat-forming species influences the diversity of associated fauna [9]. Patterns of species-level diversity and genetic diversity can be correlated, perhaps due to parallel responses to dominant environmental gradients [10] or causal relationships [11], suggesting the possibility of emergent genetic patterns at the community level. Despite these homogenizing shared forces, contrasts in life-history traits across species create differences in migration rates, habitat use and density, leading to variation in patterns of genetic diversity across co-distributed species. Life-history differences may also cause species to vary in which landscape features most strongly drive their spatial diversity patterns. There is much recent interest in investigating the major environmental correlates of population genetic patterns, partly due to the utility of protecting key landscape features or locations in conservation planning. However, prior landscape genetic studies have focused on, at most, a handful of species at a time. To date, few studies have explored the range of genetic diversity patterns and their drivers across a large sample of co-distributed species, and the role of life history in mediating these patterns and drivers [6–8,12]. None to date have focused on a sample of the ‘meta-community’ that includes representatives from multiple taxa, trophic levels and functional groups.

**Figure 1.**
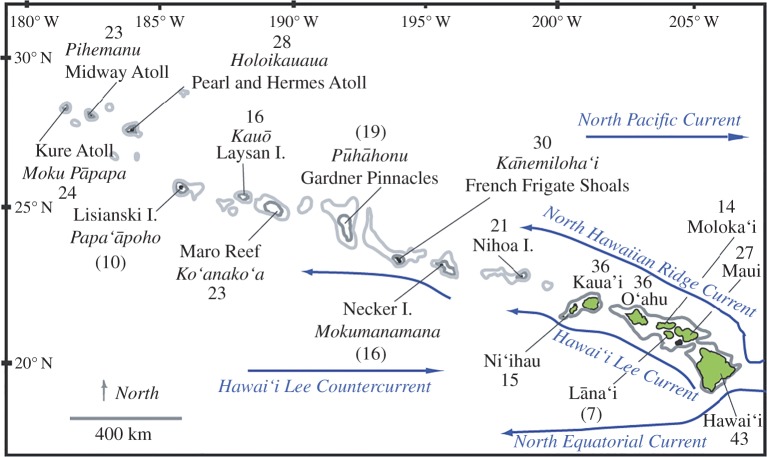
Map of the Hawaiian archipelago. Hawaiian (italics) and English (regular font) names of sampled islands and number of species genetically sampled per island are indicated; numbers in parentheses indicate islands excluded from community-level analyses of aggregate AR. Major currents are represented by arrows; 1000 and 2000 m isobaths are delineated. Islands east of 200° are the Main Hawaii Islands (MHI); islands west of 200° are the Northwest Hawaiian Islands (NWHI) and part of Papahānaumokuākea Marine National Monument.

Here, we investigate how patterns of genetic diversity vary across the Hawaiian Archipelago for a sample of 47 reef-associated animal species and ask if observed variation can be ascribed to potential seascape drivers representing benthic cover, ocean currents, habitat loss caused by sea-level change, temperature stress and other site characteristics. At first glance, the nearly linear array of discrete islands in the Hawaiian Archipelago might be expected to produce high congruence of genetic patterns as they impose uniform physical constraints on population size and dispersal distance for most reef species (figure 1). However, there are few clear cases of isolation by distance, the null expectation for genetic structure across a linear island array. Instead, patterns of population genetic structuring are highly variable, and much of this variation probably stems from the great diversity of life history and demography across reef-dwelling species [13,14]. We leveraged the large number of species genetically sampled across Hawai‘i to investigate whether species' genetic diversity patterns tend to covary with each other and with species diversity, and whether species traits account for observed variance in spatial patterns of genetic diversity and association to seascape drivers.

*A priori* hypotheses about drivers of diversity across the islands were derived from past theoretical and empirical studies. They were:

H1: Marginal populations show reduced genetic diversity due to greater isolation [15].
H2: Species richness and genetic diversity covary [4,10].
H3: Habitat size and immigration influence genetic and species diversity [16].
H4: Habitat loss during the last glacial maximum produced genetic bottlenecks that dampen diversity [7,8].
H5: The genetic diversity of habitat-forming species influences the genetic diversity of associated fauna [9,10].
H6: Ecological factors structuring reefs, namely thermal stress, coral cover and crustose coralline algae (CCA) cover, may influence genetic diversity.

We assessed these hypotheses based on both the responses of each species to the various drivers and the response of the aggregated data. The latter ‘community-level’ analysis approach treats genetic diversity of all species sampled as an emergent trait of the community, and builds on previous reports of European forest plants and Mediterranean darkling beetles which found that mean genetic diversity of a community sample shows strong correlations to landscape features and species diversity [7,12].

## 2. Material and methods

### (a) Seascape factors

Environmental and ecological data used to test our set of six hypotheses were assembled from both existing sources and new efforts. Table 1 summarizes each dataset included in analyses; see the electronic supplementary material for full descriptions. Given the small number of islands with adequate data to test our hypotheses, we sought to limit the number of seascape drivers compared in alternative model testing. Below we note our rationale for excluding additional available datasets from analysis. Estimates of benthic cover included macroalgae, sand, uncolonized, coral and CCA. We limited analyses of benthic cover type to coral and CCA cover based on established hypotheses about their roles structuring reefs. Although macroalgae also structure reefs, remote sensing methodology was unable to make the ecologically critical distinction between native and invasive types. Multi-year environmental data were available for sea surface temperature (SST), wave energy, irradiance and chlorophyll-a [17]. We limited analysis to just two metrics that are often named as drivers of coral reef communities, SST and wave energy, both available as island level averages of all grid cells within the 30 m isobath. Lastly, two habitat area measures were available—total hard-bottom area classified from satellite imagery, and bathymetric area within the 10-fathom contour. The two estimates covaried and produced identical correlation to rarefied mean AR (electronic supplementary material, table S1); we present main results using the bathymetric estimate. A principal components analysis of variation in seascape factors across islands shows that approximately 60% of the variation occurs along two axes, with the primary axis most closely associated with fish species richness, coral cover and thermal stress (electronic supplementary material, figure S1).

**Table 1.** Summary of seascape effects on a reef community. Hypothesis column indicates which numbered hypothesis was tested with the dataset; symbols **+** and − indicate whether the predicted relationship to genetic diversity is positive or negative. Support column: Y indicates support at *p* < 0.05 for the hypothesis based on regression analyses of composite genetic diversity of mtDNA data (left), nucDNA data (middle) and linear mixed modelling (right). Italics indicate factors not included in multiple regressions due to colinearity or data gaps. LGM is last glacial maximum; CCA is crustose coralline algae.

seascape driver	hypothesis	support	brief description
*fish species richness*	H2 **+**	Y Y N	Bootstrapped ‘Chao’ estimates of species counts collected on underwater visual transects conducted by NOAA's Coral Reef Ecosystem Division from 2011 to 2012. Number of sites surveyed per island was roughly proportional to reef area. Values were natural log transformed.
*coral species richness*	H2 **+**	N N N	Same as for fish species richness except that survey data spanned years 2006–2010.
habitat area	H3 **+**	Y Y Y	Log_10_ (*x* + 1) transformed estimates of total shallow-water area within the 10-fathom depth curve of each island.
potential larval immigration	H3 **+**	N N N	In-coming centrality metric calculated from modelled larval connectivity estimated from an oceanographic biophysical model parametrized for the species and habitat array.
nearest-neighbour distance	H3 −	N N N	Path distances in km between approximate centroids of islands estimated using Google Earth. The shortest distance to a neighbouring island was selected.
LGM habitat loss	H4 −	Y N Y	Estimate of the relative severity of population bottlenecks due to habitat loss 18 000 years ago during the LGM when sea level was 120 m lower; one minus the ratio of LGM to present-day habitat area.
*M. capitata genetic diversity*	H5 **+**	N N N	Rarefied AR averaged across five microsatellite loci of the coral *Montipora capitata* that each showed Hardy–Weinberg Equilibrium (non-significant tests that *F*_IS_ differs from zero).
coral cover	H6 **+**	Y Y Y	Number of pixels with more than 10% cover in IKONOS satellite imagery covering 0–30 m depth range. Expressed as a percentage by dividing by total number of pixels analysed.
CCA cover	H6 **+**	Y N Y	Same as for coral cover.
thermal stress	H6 −	Y Y N	Frequency of hotspot events, as defined by NOAA's Coral Reef Watch, when SST exceeded the maximum monthly mean temperature, over years 1985–2000, and measured at 4 km.
*wave disturbance*	H6 −	N N N	Yearly average of maximum monthly mean wave energy over years 1997–2010, measured at 1°.

### (b) Genetic diversity metrics

Genetic data assembly is detailed elsewhere [14]. Existing genetic data from samples of reef species collected in Hawaiian waters were included here for species with a minimum of six specimens each taken from of two or more islands (electronic supplementary material, dataset S1). Mean sample size per species per island was 32; 10% of the species-site samples contained fewer than 10 individuals. Excluding samples based on 6–10 specimens had insubstantial effects on results (electronic supplementary material, table S2). Analyses primarily focused on mitochondrial sequence data because only a few species have nuclear datasets; however, we also used the 11 nuclear multi-locus datasets available to assess sensitivity of some results to the marker type (electronic supplementary material, dataset S2). In addition, microsatellite data for the coral *Montipora capitata* [18] was used as a predictor variable to test hypothesis H5. For all genetic datasets, rarefied allelic richness (AR) was calculated for each island using HP-Rare [19]. As a count of alleles, AR is unaffected by other locations but possibly influenced via gene flow [20].

Multi-species ‘composite’ AR means were calculated for each island based on AR values of all species sampled. To reduce sampling bias, composite AR was rarefied to a uniform size of 12 species per island by sampling species without replacement 500 times (electronic supplementary material, dataset S3). Bias in marker composition across islands was checked and found to be minimal (electronic supplementary material, ‘Methods’). Lisianski, Kaho‘olawe and Lana‘i were excluded from analyses of composite AR due to low genetic sample sizes (*n* < 12 species). See the electronic supplementary material for extensive sensitivity analyses of this metric to sampling parameters.

### (c) Statistical analyses

Moran's I calculated with R packages *ape* and *ncf* [21] revealed no large-scale or small-scale positive spatial autocorrelation of AR across all sites, or NWHI (*p* = 0.31) and MHI (*p* = 0.99) subsets of sites. The same was true for diversity metrics of fishes (*p* = 0.16) and corals (*p* = 0.91). After assembly of all seascape data (electronic supplementary material, dataset S4), two islands were excluded from all analyses (Necker and Gardner), because they were missing data for several key seascape factors (electronic supplementary material, ‘Methods’). When using composite AR, Lisianski, Kaho‘olawe and Lana‘i were also excluded due to low sample size (*n* < 12 species), leaving 13 islands in the analysis.

Redundancy analysis (RDA) with the R package *vegan* [22] was used to visualize variation in species' correlations of AR to the seascape predictors and assess the influence of species traits on this variation. A set of species traits, summarized in table 2, was published previously [14] (electronic supplementary material, dataset S5). Pearson's *r* values describing each species's correlation of AR to each seascape factor were the dependent variables (electronic supplementary material, dataset S6). A partial RDA was also performed, using the sample size (i.e. number of islands), marker type and total marker diversity for each species as covariates, but these covariates lacked influence (electronic supplementary material, dataset S7). RDA was complemented by AIC*_c_* model selection of linear models built with species and sampling traits to determine which traits most parsimoniously explained which species showed high or low correlations of AR to seascape factors.

**Table 2.** Summary of species trait data used in analyses.

species traits	description
fish	species is fish (1) or invertebrate (0)
endemic	species is endemic to Hawai‘i (1) or widespread in the Pacific (0)
PLD	pelagic larval duration in days, log transformed
length	maximum body length in cm, log transformed
min depth	minimum reported depth occurrence in meters, log transformed
depth range	maximum depth minus the minimum depth, log transformed
habitat specialist	species is tied to particular reef features (1) or found on most reef types (0)
*θ*_ST_	strength of inter-island genetic differentiation
*F*_CT_	strength of regional genetic differentiation (i.e. groups of adjacent islands)

Congruence of spatial patterns of AR across species was gauged by Pearson's correlation coefficient for each species's AR spatial pattern regressed against the composite AR pattern of all species, for species sampled at more than five islands (*n* = 34). A one-sided *t*-test indicated whether species tended to positively correlate with composite AR, to assess a community-level trend towards congruence as in [7]. Sensitivity of congruence to sampling was assessed (electronic supplementary material, ‘Methods’). To assess the roles of life-history, sampling and genetic traits on congruence, these Pearson's *r* values were regressed against species traits in table 2 in linear AIC*_c_*-based model selection using with JMP Pro v. 11.

The same model selection procedure was also used to assess relationships of composite AR to physical and ecological seascape factors in table 1. For comparison, a similar model selection procedure was implemented for the individual AR values for all species-by-marker-by-island combinations available (*n* = 421) using a linear mixed model which designated the species-by-marker label as a random effect (electronic supplementary material, dataset S8). The latter tests the aggregated response of individual species, which does not have to be the same as the response of the aggregated data (composite AR)*.* Variation was high in AR values across species, and in which species were sampled across islands; the two modelling approaches address these issues differently but otherwise test the same hypothesis with the same response variable. The composite mean uses rarefaction to help standardize sampling variance across islands. The mixed modelling approach uses the species-level data to incorporate the variance across species and also the possibility that each species is drawn from its own distribution. Because of the large increase of parameters that need to be estimated, power is lower, but assumptions are fewer.

For both model selection procedures, data gaps for coral species richness and *M. capitata* genetic diversity estimates required omitting these predictors from model selection. Fish and coral species richness and wave disturbance were omitted due to colinearity with other factors (electronic supplementary material, ‘Methods’; electronic supplementary material, table S1). Models were limited to one to three terms for model comparison to reduce model number given the small sample size of composite AR (*n* = 13 islands). Top models were defined as ΔAIC*_c_* < 2.0, where ΔAIC*_c_* is the difference in AIC*_c_* score from the model with the minimum observed AIC*_c_* score. Model selection was repeated for regional subsets (i.e. seven islands in the NWHI and six in the MHI), motivated by the many differences between these regions that might produce distinct population genetic and ecological dynamics.

## 3. Results

### (a) Spatial diversity patterns

Spatial patterns of genetic diversity varied considerably across the 34 well-sampled species (sampled at more than four islands). For eight species sampled with both nuclear and mitochondrial markers at 6–12 islands, spatial patterns positively correlated between the marker sets, suggesting single-marker spatial trends are interpretable (electronic supplementary material, table S3). Furthermore, individual species' mtDNA AR patterns showed significant tendency for positive correlations to the composite mean AR pattern calculated using all 47 species (*t*-test 2.4, d.f. = 33, *p* = 0.01; electronic supplementary material, figure S2). Congruence retained significance when highly influential species were omitted, either because they were particularly well sampled or most positively correlated to the mean pattern (electronic supplementary material, ‘Methods’). Species with shorter PLD tended to have higher congruence (adj. *r*^2^ = 0.13, *p* = 0.04). Composite AR of all species ranged 3.0–3.8 haplotypes per six individuals across islands, and tended to show higher values at both margins of the island chain (quadratic *r*^2^ = 0.45, *p* = 0.05; electronic supplementary material, figure S3), contrary to our first hypothesis that a stepping stone habitat array produces lower diversity at the margins due to reduced immigration. Importantly, habitat area ranges three orders of magnitude across the sampled islands (6.6–470 km^2^), with large areas at the margins. High genetic diversity at the margins was also seen for the nucDNA version of composite AR of 10 species, because mtDNA and nucDNA composite AR values significantly correlated (*r*^2^ = 0.51, *p* = 0.01; electronic supplementary material, table S1). A population genetic simulation reproduced the patterns of high diversity at the margins when effective population sizes were made to vary across demes with the same relative magnitude seen in habitat area across the Hawaiian Archipelago (i.e. approx. threefold; electronic supplementary material, ‘Methods’ and figure S4).

### (b) Species-genetic diversity correlation

Estimated number of fish species (standardized for sampling effort) ranged 98–152 across islands and showed a negative quadratic fit with latitude, such that composite AR and fish species diversity significantly covaried (*r*^2^ = 0.51, *p* < 0.01; electronic supplementary material, figure S3), supporting our second hypothesis. Coral species richness ranged from 10 to 25 per island and positively correlated with fish species richness (*r*^2^ = 0.30, *p* = 0.04; electronic supplementary material, table S1), but was not significantly correlated to composite AR (*r*^2^ = 0.27, *p* = 0.08; electronic supplementary material, figure S3). Functional group specificity of species-versus-genetic diversity correlations may contribute to the mixed result [11]. Composite nucDNA AR showed almost identical relationships to species richness as mtDNA (electronic supplementary material, table S1). Considering species' individual responses, model selection showed that herbivores tended to respond positively to coral species richness and negatively to fish species richness (table 3). An obligate corallivore, *Chaetodon lunulatus*, was the species whose AR pattern most strongly correlated to coral species richness (*r*^2^ = 0.99; electronic supplementary material, dataset S6).

**Table 3.** Which species respond to which seascape drivers? Top models from multiple regression model selection built with species’ traits (independent variables) and correlation coefficients for each species’ AR regressed against individual seascape factors (dependent variable). Depth indicates depth range. (−) indicates negative relationships; all others are positive. *p < 0.05; **p < 0.01.

seascape factor	species trait predictors	Adj. *r*^2^	*K*	AIC*_c_*	*w_i_*	coeff. 1	coeff. 2
habitat area	*ϕ*_ST_	0.21**	2	27.9	0.48	6.15**	
coral cover	depth, *ϕ*_ST_	0.24**	3	18.8	0.54	0.40**	4.86*
potential larval immigration	depth, *ϕ*_ST_	0.29**	3	26.2	0.76	0.29*	5.77*
wave disturbance	depth (−), *ϕ*_ST_	0.28**	3	38.2	0.35	−0.45**	−6.25*
CCA cover	depth, count	0.30**	3	2.00	0.24	0.24*	−0.09**
LGM habitat loss	PLD (−), taxonomy^a^	0.46**	5	23.3	0.30	−0.47**	−0.79**
	marker^b^, depth (−)			34.2		0.53**	−0.002*
nearest-neighbour	*F*_CT_	0.13*	2	39.8	0.45	5.68*	
fish spp. richness	herbivore (−)	0.15*	2	34.6	0.23	−0.27*	
coral spp. richness	herbivore, *F*_CT_	0.29**	3	22.0	0.31	5.63**	−0.28*
thermal stress	no significant model						

^a^Invertebrates tend to show more negative correlation to LGM habitat loss than fishes.

^b^Cyt B dataset tend to show more positive correlation to LGM habitat loss than other marker types.

### (c) Effects of species traits on seascape drivers of genetic diversity

RDA showed that the 12 species traits together explained 49% of the variation in how species related to the seascape drivers (*p* = 0.04; electronic supplementary material, figure S5). Overall, species with stronger genetic structuring (i.e. pairwise differentiation between adjacent islands or groups of islands) showed stronger relationships to most drivers, and especially to habitat-related factors. The strongest trends in seascape associations were tied to the combined effects of taxonomy, habitat area and depth range. For example, species that showed the strongest response to habitat loss during the last glacial maximum tended to be invertebrates with shallow depth ranges, short PLD and low marker diversity, which typically indicates a past bottleneck. Shallowest species responded negatively to wave disturbance, and larger depth range was also associated with influence of coral cover and CCA cover on genetic diversity (table 3).

Interestingly, the few species that showed strong positive correlation of genetic diversity to simulations of potential larval immigration were deep-water, more genetically structured species, a rare combination in our dataset. The larval immigration model, which scaled larval production to habitat area, produced a strong peak in potential immigration at the centre of the chain (electronic supplementary material, figure S6). This pattern is expected by the stepping stone model and by the pattern of habitat area, but in conflict with the hypothesized positive effect of immigration on observed diversity, which showed high values at the margins. Larval inputs from Hawai‘i's nearest neighbour, Johnston Atoll, an alternative potential cause of the uptick in diversity at the margins, is unlikely according to the biophysical transport model (electronic supplementary material, figure S6), as are larval inputs from farther away, which were not included. Estimates of potential larval immigration pattern were also relatively insensitive to PLD and spawning seasonality.

### (d) Community-level seascape genetic analysis

A mixed modelling approach to assess the aggregated response of individual species to the seascape identified various combinations of habitat area, LGM habitat loss and coral cover as the most influential seascape correlates (table 4). Pairwise colinearity of habitat area, coral cover, thermal stress and LGM habitat loss at the 13 islands was not high (*r* < 0.5; electronic supplementary material, table S1). Model selection of composite AR provided nearly identical assessments of top drivers to the mixed modelling approach, although *p*-values differed. Pairing habitat area with coral cover, thermal stress or LGM habitat loss created competing top bi-variate models that explained 59% of the variation in composite AR across the archipelago (table 5). When limited to Northwestern Hawaiian Islands (NWHI), habitat area explained 67% of the variation in composite mtDNA diversity, whereas in the Main Hawaiian Islands (MHI), coral cover was a more parsimonious model and explained 61% of variation in mtDNA AR (table 5). Overall, positive correlation between genetic diversity and habitat area appears to be the strongest community-level seascape genetics relationship. Interestingly, equal numbers of species showed positive and negative correlations with habitat area individually. When those species with the highest positive individual correlations to habitat area are removed from the composite mean, the strong positive effect of habitat area on composite diversity remains and exceeds any single species's correlation to habitat (electronic supplementary material, figure S7).

**Table 4.** Top seascape mixed models of species AR (*n* = 421). All models for which ΔAIC*_c_* ranged 0–2 are listed (excluding models for which coefficient signs opposed our hypothesized relationships). Arch. is archipelago-wide, *K* is the number of parameters, *w_i_* is the Akaike weight. Coefficients are ordered by predictor order. ***p ≤ 0.10, *p ≤ 0.05, **p ≤ 0.01.

region	seascape predictors	*K*	AIC*_c_*	ΔAIC*_c_*	*w_i_*	coeff. 1	coeff. 2	coeff. 3
Arch.	habitat area, LGM habitat loss	4	1167.7	0	0.16	0.20**	−0.29*	
Arch.	habitat area, coral cover	4	1168.9	1.2	0.09	0.14***	0.43*	
Arch.	habitat area, coral cover, LGM habitat loss	5	1169.2	1.5	0.07	0.18*	0.21	−0.22
Arch.	coral cover	3	1169.3	1.6	0.07	0.49*		
Arch.	habitat area, CCA cover, LGM habitat loss	5	1169.6	1.9	0.06	0.19*	3.54	−0.28*
NWHI	habitat area	3	601.5	0	0.08	0.10		
MHI	coral cover, CCA	4	634.2	1.1	0.08	0.61*	23.6*

**Table 5.** Top seascape models of composite AR (*n* = 13). All models for which ΔAIC*_c_* ranged 0–2 are listed. Adj. is adjusted, other abbreviations and notation as in table 4.

region	seascape predictors	Adj. *r*^2^	*K*	AIC*_c_*	ΔAIC*_c_*	*w_i_*	coeff. 1	coeff. 2
Arch.	habitat area, coral cover	0.59**	3	−1.7	0	0.13	0.24*	0.56***
Arch.	habitat area, LGM habitat loss	0.59**	3	−1.5	0.2	0.12	0.30*	−0.29***
Arch.	habitat area, thermal stress	0.58*	3	−1.3	0.4	0.11	0.21	−0.07***
Arch.	habitat area	0.44*	2	−1.2	0.5	0.10	0.30*	
Arch.	CCA cover, thermal stress	0.56*	3	−1.0	0.8	0.09	20.6***	−0.09*
Arch.	thermal stress	0.39*	2	−0.9	0.8	0.09	−0.1*	
NWHI	habitat area	0.67*	2	3.9	0	0.83	0.27*	
MHI	coral cover	0.61***	2	11.1	0	0.52	1.02***	

## 4. Discussion

This study breaks new ground in exploring how relationships between genetic diversity and seascape variables change when examined at the species and community levels. Variation in seascape relationships across species is high, as expected of a marine meta-community with diverse life histories and tendency for high gene flow. Nevertheless, there are logical ways in which species traits predict which seascape factors most influence a given species. Species with higher spatial genetic structure showed stronger links to seascape metrics, because they are demographically more sensitive to the local environment compared with species with genetic stocks spanning many islands. Herbivores responded positively to species richness of corals, which provide shelter and algal substrate, and negatively to fish species richness, perhaps due to competition and predation. Shallower species showed stronger effects of both wave energy, where effects are concentrated, and past bottlenecks because shallow areas fluctuate considerably with sea level compared with deeper areas [23]. The lingering signal of historical habitat loss corroborates evidence for genetic bottlenecks tied to the last glacial maximum elsewhere in Polynesia, and may be most pronounced at islands with large shallow lagoons [24]. Only deep-water species with high genetic structuring, a rare combination in our dataset, showed strong influence of potential larval immigration (inferred from circulation models). The small population sizes at the many small islands in the middle of the island chain probably lead to such low diversity levels for shallow species there that even a relatively high rate of immigration cannot compensate. However, deeper species have larger and more stable habitat area even at small islands, and thus we speculate that larval immigration emerges as a detectable impact at depth, as long as genetic structuring is sufficient to create strong localized genetic responses.

### (a) Congruence and composite AR

Low-dispersal species had a tendency for higher congruence of AR, echoing previous findings for European plants where dispersal ability predicted level of congruence in genetic diversity [7]. Finding significant congruence lends support to the interpretability of composite mean genetic diversity of all species, a metric which served two purposes. First, it improved upon the traditional use of one or a few exemplar species which are often used to represent genetic diversity in reef conservation planning. The conventional exemplar species strategy would fail to uncover most of the significant driver relationships found here and, due to the variation in genetic patterns among species, would not represent much of the reef-associated community. Second, it leveraged the many single-marker datasets as replicates to overcome sampling error in assessing the role of seascape factors in shaping patterns of genetic diversity at the community level. Despite finding that species traits created some differences in how species related to seascape drivers, using mean genetic diversity did not obscure relationships to seascape drivers. Instead, the mean showed patterns that fit with most of our hypotheses. Finding the same strong correlations to habitat area and fish species richness when composite genetic diversity was calculated from 11 multi-locus nuclear datasets indicates these relationships are not marker-specific. The overall message of the results is that the composite mean seascape models show the same qualitative patterns as the mixed modelling of species-level data, and the same patterns whether it is calculated with mtDNA or nucDNA samples. Although combining data from non-homologous mtDNA loci into a single mean is not ideal, the trends across locus types are invariant, and thus the non-homologous marker dataset is probably an adequate proxy for a homologous marker dataset in this situation (i.e. with a large sample of species and an island chain). These findings support the value of continued careful synthesis of the thousands of existing ‘last-gen’ datasets in the literature, and help establish the robustness and utility of the composite mean as a tool for applied population genetics. In sum, although using a composite mean comes with accompanying errors and assumptions, it has a potentially important role to play in distilling complex data and aiding the uptake of genetic data by conservation and management.

### (b) Roles of seascape drivers

Habitat area appears to be a dominant influence on genetic diversity, with high predictive power for both genetic and species-level diversity across islands. The dominant effect of habitat area on diversity is widely understood [4]. Another logical finding was the strong influence of coral cover on reef genetic diversity. Coral cover acts as a modifier of habitat quality and quantity, by providing shelter, food and rugosity. Confirming basic relationships between diversity and habitat factors bolsters the validity of the unexpected influences of additional seascape factors at the island scale.

Finding little influence of our estimate of potential immigration on mean genetic diversity fits with the rarity of isolation by distance among these species shown previously across the Hawaiian Archipelago [14]. Interestingly, dispersal metrics had little spatial variation relative to the variation in habitat size across islands. This suggests that rate of drift may be more spatially variable than migration, and thus more influential to genetic diversity of shallow-water reef communities [25,26]. The increase in diversity at marginal locations is due to the coincidence that the largest habitat areas within the Hawaiian Archipelago are found at the margins, and this was confirmed by population genetic simulation (electronic supplementary material, figure S4). Additionally, realized immigration might differ from modelled biophysical transport perhaps due to effects of chemical cues from coral and CCA, post-settlement mortality or density-dependence affecting rates of settlement and recruitment [27,28].

Although the effect of recent thermal stress on genetic diversity was not one of the strongest seascape factors, it showed significant negative correlation to composite AR and roughly half of the species showed a moderate or strong correlation individually (electronic supplementary material, dataset S6). Thermal stress is known to directly affect health of coral and algal populations with cascading effects on reef habitat complexity, quality or productivity [29]. Low-latitude Pacific reefs have experienced thermal stress up to 240% higher than Hawai‘i and documented ecological effects have also been more severe [30], suggesting that recent declines in genetic diversity might also be more severe at lower latitudes. Indirect or synergistic effects from other predictors or unanalysed ‘latent’ factors may influence which seascape drivers show strong influence on genetics. For example, thermal stress shows moderate correlation with fish and coral species richness (electronic supplementary material, figure S1), which may partially account for its correlation to genetic diversity.

### (c) Conservation implications

The seascape relationships identified here lend crucial and novel empirical support to difficult, urgent and controversial decisions unfolding about how best to conserve genetic diversity at both community- and species-level scales. Specifically, these results lend a new form of support to the idea that conserving large, intact reefs with high coral cover protects the diversity of entire reef communities, and thus supports the emerging strategy of creating large-scale marine protected areas pursued by the Big Ocean Initiative, among others [31]. Active conservation of reef biodiversity will promote future resilience to mounting stress posed by increasing coastal development and adaptive potential to climate change [31–33]. In particular, the likely negative effect of thermal stress on genetic diversity may signal the potential for global warming to compromise the adaptive capacity and genetic integrity of not just corals but the entire coral reef community. Papahānaumokuākea Marine National Monument safeguards the majority of Hawaiian reef biodiversity, but populations in the MHI tend to be genetically distinct [13] and warrant additional protections. Our study shows that genetic diversity varies across the MHI, extending the argument for targeted protection of genetic resources of reefs throughout the MHI. Notably, Hawai‘i Island has the greatest amount of coral reef area, harbours maximal genetic diversity, on average, and probably serves a unique and particularly influential role due to its large size at the margin of the chain. Nihoa and Ni‘ihau, which sit at the transition between NWHI and MHI, also warrant particular focus for future research and protection due to intriguing combinations of high genetic divergence and unusual fish composition [34].

### (d) Eco-genetic feedbacks

It might seem puzzling that the multi-species mean showed strong positive correlation to habitat area even though equal numbers of individual species showed positive and negative correlations to habitat area (electronic supplementary material, figure S7). There are many ways that macro-ecological forces and species interactions could generate this seemingly contradictory pair of results. Note that which species are included in which islands' multi-species means is driven largely by ecology—most species do not occur commonly at all islands, and sampling efforts were in most cases exhaustive, such that the lack of DNA samples from some islands reflects the species's rarity or the absence there. The cause of such absence or rarity can not only be due to environmental mismatch (e.g. colder temperatures at the northern end of the chain), but also due to lottery/precedence effects on recruitment success or high mortality due to competitive exclusion, or intense predation.

A specific example that demonstrates the possible linkage of community ecology to community-level genetic patterns in Hawai‘i is that endemic and widespread reef fishes exhibit opposite linear gradients in density across the archipelago, both in aggregate and for pairs of endemic and non-endemic congeners [34]. Whereas the density ratio of endemics to non-endemics is 2 : 3 at the southeastern end of the chain, it flips to 3 : 2 at the northwestern end, with an inflection point of 1 : 1 around French Frigate Shoals at the midpoint. The environmental climate of the northwestern end of the chain is unusual for Pacific coral reefs, and endemics show a competitive advantage over non-endemics under these conditions. Our genetic samples also showed an increase in proportion of endemics with latitude that was driven by the sampled fishes. Assuming genetic diversity scales with numerical abundance and only abundant species are genetically sampled, this swapping of competitive dominance across islands would lead to the same calculations of mean AR despite individual species showing very different spatial patterns of AR. In support of this possibility, we found that for the 10 endemic fishes in our dataset the mean correlation of genetic diversity to habitat is negative (*r* = −0.06), while for the 13 non-endemic fishes it is positive (*r* = 0.11). Nevertheless, other processes aside from endemism probably also contribute to the emergent trends in composite diversity.

As a whole, community-level genetic diversity reflects both the bottom-up result of each species's population genetic history, as is well known and understood, but also top-down influences of community filtering of species composition and interspecific constraints on the composite effective population size of the species assemblage. In other words, although species-level processes filter and constrain community-level genetic patterns, community-level processes also filter and constrain community-level genetic patterns. These latter effects are as yet not well documented and studied. However, recent demonstration that haplotype turnover closely tracks species turnover supports the idea that mean genetic diversity is constrained by macro-ecological forces in addition to well-understood species-level processes (e.g. genetic drift) [35].

### (e) Composite genetic diversity as an emergent property?

It is interesting to consider how and why composite diversity can show higher correlation to habitat than any species shows individually. If the boost in correlation is entirely due to the large sample size (i.e. treating species as locus replicates), this suggests that all species would show the same high correlation to habitat area with better genomic sampling. However, it is likely that some fraction of species in a community will always show negative or no correlation with area due to particular species traits, species interactions and historical effects. Indeed, a recent synthesis of species–genetic diversity correlations finds both positive and negative relationships are prevalent at the species level [36]. We hypothesize that composite genetic diversity is likely to be an emergent property at the community level that shows responses to the seascape distinct from individual species' responses, due to the aforementioned linkage to other community-level traits such as species composition and diversity. The distinction between drivers of individual species' genetic diversity and drivers of multi-species genetic diversity is analogous to the distinction between drivers of abundance patterns within a species and drivers of total abundance of individuals within a community, which is well understood to be an emergent ecological property of the community. Mathematically, when species composition, species richness and/or species abundance distributions vary strongly across space, composite genetic diversity should diverge more strongly from representing the mean genetic diversity of individual species, but continue to mirror species diversity patterns. A mechanistic macro-ecological link between species and genetic diversity patterns exists if the same large-scale assembly rules that dictate species composition also influence composite genetic diversity [37]. Specifically, a species's rarity and small range size lead to low total gene diversity, and also lead to greater chance of local extinction, thereby impacting both composite genetic diversity and species composition. Further work is needed to better understand these sorts of feedbacks that link composite genetic diversity to species abundance distribution, community assembly and its drivers. Detecting macro-ecological regularities requires large sample sizes; using the multi-species mean overcomes stochasticity associated with any single dataset so that the most dominant pattern and its drivers are more powerfully assessed [12]. The composite mean may be a convenient and appropriate tool that can reveal underlying macro-ecological processes influencing diversity at all hierarchical levels, and reveal these effects more strongly than a comparison of the patterns of individual species. The findings as a whole demonstrate that continued expansion of integrative studies of community-level genetic diversity holds promise to elucidate the complex interdependencies across biodiversity levels and provide critical information to stem the loss of global biodiversity.

## Supplementary Material

Supplementary Material

## Supplementary Material

Supplementary Data Spreadsheets

## Data accessibility

Data have been deposited at DRYAD (http://dx.doi.org/10.5061/dryad.qm288) and BCO-DMO (http://www.bco-dmo.org/project/552879).
