# Supplementary material for: The DNA of coral reef biodiversity: predicting and protecting genetic diversity of reef assemblages
Source: Proc Biol Sci. 2016 Apr 27;283(1829):20160354. doi: 10.1098/rspb.2016.0354 (PMC4855387; doi:10.1098/rspb.2016.0354)
Supplement: Supplementary Material [file rspb20160354supp1.docx]

**Contents**

**Page 2: Appendix 1** - Contributors to the Hawaiʻi Reef Connectivity Consortium

**Page 4: Supplementary Methods concerning input data**

**Page 7: Sensitivity Analyses Methods and Results**

**Page 10: Literature Cited**

**Page 12: Table S1 -** Colinearity matrix of all input data

**Page 13: Table S2** - Effect of minimum sample size of 6 vs. 10 on main results

**Page 14: Table S3** – Correlation of nuclear and mtDNA spatial patterns for 8 species

**Page 15: Figure S1** – Principal Components Analysis of seascape factors

**Page 16: Figure S2** – Distribution of congruence values across species

**Page 17: Figure S3** – Plots of composite mtDNA diversity against 12 factors

**Page 18: Figure S4** – Output of population genetic simulations

**Page 19: Figure S5** – Redundancy Analysis tri-plot

**Page 20: Figure S6** – Modeled larval immigration map and latitudinal pattern

**Page 21: Figure S7** – Distribution of species AR correlation to habitat area

**Page 22: Figure S8** – Correlations of composite AR to fish species richness

**Appendix 1**

*Names and addresses of 31 contributors to the Hawaiʻi Reef Connectivity Consortium group authorship. Consortium members contributed existing data to the study, and verified proper data usage and interpretation of results.*

Kimberly Andrews, Dept Fish and Wildlife Sciences, University of Idaho, 875 Perimeter Drive MS 1136, Moscow ID 83844-1136

Iliana Baums, Department of Biology, The Pennsylvania State University, 208 Mueller Laboratory University Park, PA, 16802, USA

Moisés A. Bernal, California Academy of Sciences, 55 Music Concourse Drive, Golden Gate Park, San Francisco, CA 94118, USA

Giacomo Bernardi, Department of Ecology and Evolutionary Biology, University of California Santa Cruz100 Shaffer Road, Santa Cruz, California, 95060

Christopher Bird, Marine Biology Program, Department of Life Sciences, Texas A & M University–Corpus Christi, 6300 Ocean Drive, Corpus Christi, Texas 78412, USA

Holly Bolick, Bishop Museum, 1525 Bernice St, Honolulu, HI, 96817, USA

Brian Bowen, Hawaiʻi Institute of Marine Biology, University of Hawaiʻi, Kāneʻohe, HI 97644, USA

Richard Coleman, Hawaiʻi Institute of Marine Biology, University of Hawaiʻi, Kāneʻohe, HI 97644, USA

Gregory T. Concepcion, Pacific Biosciences, 1380 Willow Rd, Menlo Park, CA, 94025, USA

Matthew T. Craig, Southwest Fisheries Science Center, National Marine Fisheries Service, National Oceanic and Atmospheric Administration, 8901 La Jolla Shores Drive, La Jolla, CA, 92037, USA

Toby S. Daly-Engel, Department of Biology, University of West Florida, 11000 University Parkway, Pensacola, FL, 32514, USA

Joseph D. DiBattista, Department of Environment and Agriculture, Curtin University, PO Box U1987, Perth, WA 6845, Australia

Jeffrey Eble, Center for Environmental Bioremediation and Diagnostics, University of West Florida, 11000 University Parkway, Pensacola, FL, 32514, USA

Iria Fernandez-Silva, California Academy of Sciences, 55 Music Concourse Drive, Golden Gate Park, San Francisco, CA 94118, USA

Erik C. Franklin, Hawaiʻi Institute of Marine Biology, School of Ocean and Earth Science and Technology, University of Hawaiʻi, Kāneʻohe, HI 97644, USA

Alan Friedlander, Fisheries Ecology Research Lab, Department of Biology, University of Hawaiʻi, Honolulu, Hawaiʻi 96822, USA and Pristine Seas-National Geographic, Washington DC 20036, USA.

Michelle R. Gaither, School of Biological and Biomedical Sciences, Durham University, South Road, Durham DH1 3LE, UK *and* California Academy of Sciences, Ichthyology, 55 Music Concourse Drive, San Francisco, CA 94118, USA

Jamison Gove, Ecosystems and Oceanography Division, Pacific Islands Fisheries Center, National Oceanographic and Atmospheric Administration Inouye Regional Center, Honolulu, HI, 96818

Mathew Iacchei, Department of Oceanography, University of Hawaiʻi at Mānoa, 1000 Pope Rd., Honolulu, HI, 96822, USA

Yanli Jia, International Pacific Research Center, University of Hawaiʻi at Mānoa, Honolulu, HI, 96822, USA

Donald Kobayashi, Ecosystems and Oceanography Division, Pacific Islands Fisheries Center, National Oceanographic and Atmospheric Administration Inouye Regional Center, Honolulu, HI, 96818

Nicholas R. Polato, Department of Ecology & Evolutionary Biology, 215 Tower Rd., Cornell University, Ithaca, NY 14853, USA

Malia Ana J. Rivera, Hawaiʻi Institute of Marine Biology, University of Hawaiʻi, Kāneʻohe, HI 97644, USA

Luiz A. Rocha, California Academy of Sciences, 55 Music Concourse Drive, Golden Gate Park, San Francisco, CA 94118, USA

Joshua Reece, Valdosta State University, 1500 North Patterson Street, Valdosta, GA 31698, USA

Derek Skillings, Hawaiʻi Institute of Marine Biology, University of Hawaiʻi, Kāneʻohe, HI 97644, USA

Scott R. Santos, Department of Biological Sciences, Auburn University, Auburn, AL 36849, USA

Zoltan Szabo, Hawaiʻi Institute of Marine Biology, University of Hawaiʻi, Kāneʻohe, HI 97644, USA

Molly Timmers, Hawaiʻi Institute of Marine Biology, University of Hawaiʻi, Kāneʻohe, HI 97644, USA

Lisa Wedding, Center for Ocean Solutions, Stanford University, 99 Pacific Street, Suite 555E, Monterey, CA 93940

Gareth J. Williams, Center for Marine Biodiversity and Conservation, Scripps Institution of Oceanography, La Jolla, CA 92083, USA

Nicholas M. Whitney, Behavioral Ecology and Physiology Program, Mote Marine Laboratory, Sarasota, FL 34236

**Supplemental Methods concerning input data**

**Assembly of existing seascape datasets.** ‘Habitat area’ used Log_10_ (x+1) transformed estimates of total shallow-water area within the 10-fathom depth curve of each island [[1](#_ENREF_1)]. An alternative estimate of habitat area, the total hard-bottom area classified from satellite imagery (see below), strongly correlated with this metric and produced identical correlation to rarefied mean AR as the bathymetric estimate (Table S1).

Percentages of ‘Coral cover’ and ‘Crustose coralline algal (CCA) cover’ used published estimates produced from IKONOS satellite imagery covering 0-30m depth range in shallow-water coral reef ecosystems [[2](#_ENREF_2), [3](#_ENREF_3)]. Grid cells were classified as one of six benthic cover types when at least 10% of the grid cell contained a single cover type (i.e., hard coral, CCA, ‘uncolonized hard bottom,’ macroalgae, sand and ‘hard bottom indeterminate cover’). In the MHI, finer subcategories of these six types were combined to match NWHI classes. Necker was excluded from analyses of benthic cover because of large classification gaps. Total area metrics were converted to percentage cover by dividing number of typed grid cells by the total grid cells classified at each island.

Multi-year environmental data were available for sea surface temperature (SST), wave energy, irradiance and chlorophyll-a as island level averages of all grid cells within the 30m isobath [[4](#_ENREF_4)]. ‘Thermal stress’ represented the frequency of Hotspot events, as defined by NOAA’s Coral Reef Watch, when SST exceeded the maximum monthly mean temperature over years 1985-2000, measured at 4 km. ‘Wave disturbance’ represented yearly average of maximum monthly mean wave energy over years 1997-2010, measured at one degree.

During the last glacial maximum (LGM) 18,000 years ago, sea level was as much as 120 m lower than present [[5](#_ENREF_5)], severely restricting coral reef habitat in some parts of the archipelago. ‘LGM habitat loss’ compared current bathymetric area within 0-45 m depth and area within 120-165 m depth, which is the analogous depth range when sea level was 120m lower during the LGM [[6](#_ENREF_6)]. Necker and Gardner Pinnacles were excluded because unusual bathymetry at these pinnacles makes this approach uncertain. To estimate the likely severity of population bottlenecks due to habitat loss during the LGM, we subtracted from one the ratio of LGM to present day area estimates.

Distance to nearest island was used as a simple metric of probable connectivity. ‘Nearest neighbor distance’ was estimated on GoogleEarth using path distances between approximate centroids of islands and selecting the shortest distance to a neighboring island. A second, bio-physical metric of connectivity is described below.

**Larval Dispersal Model:** A particle tracking transport model based simulated larval dispersal using a regional implementation of the Massachusetts Institute of Technology general circulation model [[7](#_ENREF_7)] (MITgcm) for years 2009-2014 configured for the Hawaiian Archipelago (175°E-210°E and 15°N-35°N), with hourly output at 4 km horizontal resolution across 50 vertical layers. The model was forced by surface wind stress at a resolution of 0.25 degree from satellite scatterometer measurements for the period of May 2009 to May 2014 at daily intervals, using monthly climatological surface heat and fresh water fluxes, and ocean conditions from a global ocean estimation system at 8 km resolution at the lateral open boundaries. Reef habitat was defined for 687 pixels, representing all shallow reefs and submerged banks present in the Archipelago, using a combination of IKONOS-derived data [35, 36] and modeled coral distributions in the MHI [[8](#_ENREF_8)]. The relative amount of larvae released per reef cell was determined by the percentage of reef habitat in each pixel. Larvae were released every 2 weeks for a maximum pelagic larval duration (PLD) of 45 days, an approximate median duration from the range of PLD known for our genetically sampled species and the majority of common reef taxa [[9](#_ENREF_9)]. Relative dispersal rates in Hawaii were insensitive to PLD beyond 40 days.

Larval dispersal estimates were compared from two alternative biophysical modeling approaches, an advection transport algorithm and an individual-based model (IBM), that both used the MITgcm data, the same habitat designations, and a 45-day maximum PLD bounded to 3 – 33 m depth. For the IBM, 50 particles were released each day (totaling 1673 days) from each habitat pixel, creating >57 million particles total/run. Particles were kept at 50 meters depth, allowed to passively disperse for 45 days without directional behavior. Particles that reached <5 km distance from defined reef pixels after 45 days were deemed settled and tallied by island. Results were averaged at monthly resolution for all release dates. For the second larval dispersal simulation, an advection transport algorithm [[10](#_ENREF_10), [11](#_ENREF_11)], dispersal kernels were resolved to densities of < 1/60 million larvae per km^2^, allowing rare and/or infrequent events to be quantified. For both models, outputs were compared when averaged across all release dates, and when limited to release dates April through July, a window when the majority of the species in the genetic dataset spawn and settle. The results were highly correlated for release dates and for the two alternative modeling approaches. Thus only the data produced by the advection transport model for all months was used further.

A single metric from this output was used in analysis to best represent ‘potential larval immigration’: an estimate of in-closeness centrality, which measures the relative distances each node (island) is from all other nodes based on dispersal to that node (i.e., immigration) [[12](#_ENREF_12)]. High closeness means that a site has strong, direct connections to many other sites instead of indirect or no connections. Exploratory analysis of other metrics from the model output, such as number of settlers, self-recruitment rate, betweeness centrality and out-closeness centrality revealed no further insights about mean genetic diversity.

**Species richness datasets.** ‘Fish Species Richness’ and ‘Coral Species Richness’ estimates came from coral and fish species counts collected on underwater visual transects by the Pacific RAMP, conducted by the NOAA Pacific Islands Fisheries Science Center’s Coral Reef Ecosystem Division, from 2011-2012 for fishes and from 2006-2010 for corals. Estimates of mobile invertebrate species richness were unavailable. Fish surveys were based on a stratified random design across reef zone and depth zones of <30m depth on hard bottom habitats with a stationary point count [[13](#_ENREF_13)]. Coral surveys were conducted at permanent sites distributed haphazardly within reef zones at <30m depth on hard bottom habitats using a linear-point intercept method [[14](#_ENREF_14)]. Number of sites surveyed per island was roughly proportional to reef area. Both methods identified taxa to the finest taxonomic level possible and only observations at the species level were used in diversity calculations. Sites were pooled by island [[15](#_ENREF_15)] and incidence-based bootstrapped ‘chao’ estimates were calculated with the *specpool* function in the R package *vegan* [[16](#_ENREF_16)]; this procedure corrects for varying numbers of transects per island.

**Colinearity of seascape predictors:** Before model selection, colinearity between pairs of predictor variables was assessed with significance testing of linear and quadratic regression, and principal components analysis (Fig. S1). Regression tests showed covariance of latitude, coral cover and wave disturbance to be the most prominent concern. Wave disturbance increases linearly with latitude with near-perfect correlation. Coral cover declines linearly with latitude (and waves) until an asymptote north of 24 ˚N, creating a strong curvilinear relationship. Therefore of the three predictors, we include only coral cover in all analyses because its positive correlations with both mean genetic diversity and fish species richness are more interpretable than the apparent bowl-shaped relationships of latitude and wave disturbance to diversity metrics (Fig. S5). However, latent effects of latitude and/or wave disturbance may confound interpretation of the relationship between coral cover and mean genetic diversity. Also, the co-variation of fish species richness, coral cover and thermal stress make the detected effects ascribed to any one of these factors potentially augmented effects from the other two (Fig. S1).

Other latitudinal gradients in the predictor data are noted below. LGM habitat loss showed a significant negative quadratic relationship with latitude, caused by four extreme low values at the archipelago’s margins (r^2^=0.64, p=0.004). We repeated analyses involving LGM habitat loss without these sites (Hawaiʻi, Maui, Molokaʻi and Kure) and found that significance of its relationship to genetic diversity were identical, so a latent effect of latitude is unlikely. Thermal stress showed a moderate positive quadratic fit to latitude and highest values at Lisianski, the epicenter of observed coral bleaching in the NWHI in 2002 [[17](#_ENREF_17)]. Because the oceanographic mechanism producing this gradient is unknown [[17](#_ENREF_17)] and thermal stress showed no linear or quadratic relationship to any other predictors, we retained this predictor. The remaining predictors, Habitat area, CCA cover, and *M. capitata* genetic diversity showed no significant linear or quadratic colinearity (i.e., Pearson’s r>0.7; Table S1).

**Linear modeling of allelic richness:** Although Poisson-distribution with log link function and an over-dispersion parameter are often used in GLMs of high-variance count data (ie., richness), Poisson model fits and P values were identical to normal-distributed GLMs with identity link functions (i.e., ordinary linear models), so AIC-based model selection used the latter. Indeed, because the species richness and mean allelic richness data used here have no zeros and are means of count data, their distributions are expected to be symmetric and bell shaped, albeit limited to positive values. Species Richness data was natural log transformed. For all GLM models, all pairwise relationships, interactions, and residuals of multiple regressions were inspected to determine appropriate treatment, and residuals were tested for spatial autocorrelation.

**Simulation of observed spatial pattern of composite genetic diversity.** We simulated a linear stepping-stone model at equilibrium consisting of 12 populations and compared two scenarios: (1) all populations had equal effective sizes, and (2) the two marginal populations were three times larger than all other populations. Simulations were run with *Fastsimcoal2.5* [18]. Results are based on 200 replicates of each scenario and sample size of 10 haploid individuals. We simulated mtDNA sequences 1kb in length and mutation rate = 2*x*10^-6^. In the low-migration scenario *m* = 0.001 between neighbouring populations (i.e. total proportion of migrants in non-marginal demes was 0.002). In the high-migration scenario *m* = 0.01 (i.e. total proportion of migrants in non-marginal demes = 0.02). The results show that when migration rates are low (Fig. S3a), the effect of the reduced migration at the margins is more than compensated by the larger effective size of the marginal populations. The same overall pattern is observed for higher migration rates (Fig. S3b) but in this case, genetic diversity is more evenly distributed among demes so the pattern is less pronounced.

**Sensitivity Analyses Methods and Results**

We assessed sensitivity of results to various aspects of the study design, detailed below.

1. Marker Type - Due to existing concern about reliability of single-locus genetic data and interpretability of mitochondrial data, we sought to confirm that spatial patterns of AR based on mitochondrial data are consistent with other marker types within a species. We were able to compare rarefied AR from nuclear and mitochondrial datasets individually for 8 species with both types of markers available. Comparisons used Pearson’s r values and confirmed positive covariation (Table S2).

Eleven species total were sampled with multiple nuclear markers across the Hawaiian Archipelago (Dataset S2). This nucDNA dataset includes two coral species not included in the mtDNA dataset; all other species are included in the mtDNA dataset. We used HPRARE to calculate rarefied allelic richness for these 11 datasets, using a rarefaction size of 20 alleles (minimum sample size of 10 individuals). Twelve islands were sampled with at least 6 species, including Necker and Gardener. The number of species sampled per island for the nuclear dataset was correlated with that of the mtDNA dataset (r^2^=0.47, p=0.02). Because variation in sampled species per island was low (it ranged 6 to 11 species), rarefying the island means was not necessary. We compared the nucDNA values of mean allelic richness to the values from mtDNA. We again excluded Necker and Gardner from analyses as one or the other behaved as a strong outlier in all tests. For the remaining ten islands, the two estimates of composite genetic diversity are well-correlated (r^2^=0.50, p = 0.02) and showed similar responses to most seascape factors (Table S1). Notably, fish species richness strongly predicted composite nucDNA diversity (r^2^=0.73, p=0.002), with a stronger fit than for composite mtDNA diversity at this subset of ten islands (r^2^=0.44, p=0.02). Habitat area was also a significant predictor of composite nucDNA diversity (r^2^=0.41, p=0.05), and this was the top model in alternative model selection of univariate and bi-variate models of composite nuclear diversity. The most parsimonious bi-variate regression models were a competing set of habitat area paired with coral cover, CCA cover, thermal stress or LGM habitat loss all within ∆AIC_c_ of 2.0, a similar list to the top models for the mtDNA dataset. However, none of the secondary coefficients showed statistical significance. The smaller sample size of species and islands in the nucDNA dataset likely reduces power to pick up these secondary effects.

2. Minimum sample size - To assess sensitivity of the study’s results to the rarefaction size of 6 haplotypes per sample, we repeated all analyses for a rarefaction size of ten haplotypes, excluding samples smaller than this minimum. The inclusion of small samples doesn’t change any of the results (Table S3). The notion that polymorphic loci require large sample sizes is in fact a common misconception in the field [19-22].

3. Diversity metric - As an alternative genetic diversity metric to allelic richness, we calculated gene diversity (GD), the mean rarity of alleles and resampled island means to get a composite genetic metric. Mean GD correlated highly with mean AR (Table S1), so only AR was used in main statistical analyses.

4. MtDNA locus – In concept, pooling allelic richness data for non-homologous mitochondrial loci may seem problematic because the marker-species diversity estimates are not replicates from a single “population” but instead taken from a highly similar set of populations each representing a genome region. However, our results indicate that error associated with combining samples taken from several regions of mtDNA does not overwhelm the signal and bias is very low. Using a large sample (e.g., adding species) is a valid way to overcome noise, and the fact that we find a significant result indicates that the signal is strong using our sample. Thus, the estimates made from non-homologous nuclear and mitochondrial loci can be viewed as reliable and useful proxies for estimates made from homologous loci. We detail our analyses supporting this statement below.

Species sampled with COI showed higher marker diversities on average than the other marker types. However, this trend was driven by three COI datasets with unusually high genetic diversity- two hermit crabs and a limpet (Calcinus haigae, C. seurati and Cellana talcosa). All three species had very poor sampling, and as such these species were excluded from species-level analyses and have little impact on composite AR. Marker type had no effect on species level results in the redundancy analysis. We also confirmed that correlation coefficients for each species’ AR to the top seascape factors (habitat area, coral area, fish species richness, and thermal stress) are uninfluenced by marker type using ANOVA. Neither marker type nor gene diversity had any effect on congruence. Specifically, a t-test showed that species sampled with COI or CB were no more likely to show high or low congruence to the composite AR values than species sampled with other loci (t=1.2, p=0.22).

Bias could nevertheless arise in the composite AR results if certain islands were predominantly sampled with mtDNA regions of low diversity. Cytochrome B shows lower diversity than other mtDNA regions in our dataset. Average of total gene diversity from the sampled species is 0.66 for CytB and ~0.73 for all other markers – not a large difference. In our case, the most influential islands to the trends in patterns and drivers of composite AR are Hawaii (highest AR) and Nihoa (lowest AR), so bias in marker composition is most crucial for these two islands. In fact, 49% of species at Hawaii were sampled with Cyt B, whereas 65% of species at Nihoa were sampled with Cyt B, potentially amplifying their difference in AR. To figure out how much bias this difference in marker composition creates, we calculated the mean total genetic diversity at each location as a weighted average using the proportion of CytB markers in the island’s sample. We find the expected difference in diversity due to the different marker ratio at the two islands is 1% (mean total gene diversity of 0.696 at Hawai’i vs. 0.685 at Nihoa). In comparison, the actual difference in rarefied AR for the two locations is 22%, and the relative difference in raw mean gene diversity (more comparable to total gene diversity) is 16%. A 1% bias cannot produce a 16% effect. Thus, bias is not contributing measurably to the results.

5. Species sampled - We explored the sensitivity of the congruence results to the species included in the composite mean. Because there are 37 species in the calculation of the rarified island mean, excluding one doesn’t change the mean enough to make a difference in congruence results. We chose to test the stability of the composite mean and congruence results by jackknifing in ways to purposefully disrupt them. First, we recalculated the resampled island means after omitting, as a group, the three species with the highest congruence to the resampled mean (celexa, halorn and panpen, all with congruence r>0.7 ). Overall distribution of congruence values to the new resampled mean was nearly identical, and the positive skew of the distribution was nearly identical (with identical statistical significance p=0.03). Although the congruence of the three omitted species to the new mean was lower (r=0.45, 0.61 and 0.69), they remained some of the highest observed congruence values. We repeated this type of test a second time, omitting four other species with high influence on the results because they were collected at many islands, including islands with small total number of species sampled (acapla, ophpic,parmul,abuabd). These species showed a mix of low and high congruence (r = 0.05, 0.13, 0.44 and 0.55). Again overall distribution and skew of congruence to the new multi-species mean were unchanged when calculated without these four species, yet the four species showed no appreciable change in their individual congruence to the mean from which they were omitted (r = 0.07, 0.08, 0.39 and 0.56).

6. Taxon - We re-calculated the rarefied multi-species mean allelic richness using only fishes, to test the possibility that the fishes were driving all of the seascape relationships. Rarefaction used a sample of 8 species per island instead of 12 to accommodate lower samples at some islands. Correlation of the fish-only mean to fish species richness, habitat area and other seascape factors were very noisy due to certain islands becoming large outliers. Even with these islands excluded, the mean of all 47 species showed higher correlation to fish species richness than the mean of the 37 fishes (all: R2= 0.67, p = 0.001; fishes: R2 = 0.46, p = 0.01; Fig S8). Most likely, running analyses with fish-only means of genetic diversity, although more comparable to fish species richness estimates, reduces sample sizes at some islands to low levels, compromising statistical power of seascape analyses.

**Literature Cited**

1. Rohmann S.O., Hayes J.J., Newhall R.C., Monaco M.E., Grigg R.W. 2005 The area of potential shallow-water tropical and subtropical coral ecosystems in the United States. *Coral Reefs* **24**(3), 370-383. (doi:10.1007/s00338-005-0014-4).

2. Battista J., Costa B.M., Anderson S.M. 2007 Shallow-water benthic habitats of the main eight Hawaiian Islands (DVD). In *NOAA Technical Memorandum NOS NCCOS* (Silver Spring, MD.

3. Friedlander A., Keller K., Wedding L.M., Clarke A., Monaco M.E. 2009 A marine biogeographic assessment of the Northwestern Hawaiian Islands. In *NOAA Technical Memo NOS NCCOS 84* (p. 363. Silver Spring, MD.

4. Gove J.M., Williams G.J., McManus M.A., Heron S.F., Sandin S.A., Vetter O.J., Foley D.G. 2013 Quantifying climatological ranges and anomalies for Pacific coral reef ecosystems. *Plos One* **8**(4), e61974. (doi:10.1371/journal.pone.0061974).

5. Bard E., Hamelin B., Arnold M., Montaggioni L., Cabioch G., Faure G., Rougerie F. 1996 Deglacial sea-level record from Tahiti corals and the timing of global meltwater discharge. *Nature* **382**(6588), 241-244. (doi:Doi 10.1038/382241a0).

6. Baums I.B., Godwin L.S., Franklin E.C., Carlon D.B., Toonen R.J. 2014 Discordant population expansions in four species of coral-associated Pacific hermit crabs (Anomura: Diogenidae) linked to habitat availability resulting from sea-level change. *J Biogeogr* **41**(2), 339-352. (doi:10.1111/Jbi.12181).

7. Marshall J., Adcroft A., Hill C., Perelman L., Heisey C. 1997 A finite-volume, incompressible Navier Stokes model for studies of the ocean on parallel computers. *Journal of Geophysical Research* **102**, 5753-5766.

8. Franklin E.C., Jokiel P.L., Donahue M.J. 2013 Predictive modeling of coral distribution and abundance in the Hawaiian Islands. *Mar Ecol Prog Ser* **481**, 121-132. (doi:10.3354/meps10252).

9. Shanks A.L. 2009 Pelagic larval duration and dispersal distance revisited. *Biological Bulletin* **216**(3), 373-385.

10. Treml E.A., Halpin P.N., Urban D.L., Pratson L.F. 2008 Modeling population connectivity by ocean currents, a graph-theoretic approach for marine conservation. *Landscape Ecol* **23**, 19-36.

11. Treml E.A., Roberts J.J., Chao Y., Halpin P.N., Possingham H.P., Riginos C. 2012 Reproductive Output and Duration of the Pelagic Larval Stage Determine Seascape-Wide Connectivity of Marine Populations. *Integr Comp Biol* **52**(4), 525-537. (doi:10.1093/icb/ics101).

12. Borgatti S.P. 2005 Centrality and network flow. *Social Networks* **27**(1), 55-71. (doi:<http://dx.doi.org/10.1016/j.socnet.2004.11.008)>.

13. Williams I.D., Richards B.L., Sandin S.A., Baum J.K., Schroeder R.E., Nadon M.O., Zgliczynski B., Craig P., McIlwain J.L., Brainard R.E. 2011 Differences in reef fish assemblages between populated and remote reefs spanning multiple archipelagos across the central and western Pacific. *Journal of Marine Biology* **2011**, 14. (doi:10.1155/2011/826234).

14. Brainard R., Asher J., Blyth-Skyrme V., Coccagna E.F., Dennis K., Donovan M.K., Gove J.M., Kenyon J., Looney E.E., Miller J.E., et al. 2012 Coral reef ecosystem monitoring report of the Mariana Archipelago: 2003-2007. In *PIFSC Special Publication* (p. 1019, Pacific Islands Fisheries Science Center,.

15. Palmer M.W. 1990 The estimation of species richness by extrapolation. *Ecology* **71**, 1195-1198.

16. Oksanen J., Blanchet F.G., Kindt R., Legendre P., Minchin P.R., O'Hara R.B., Simpson G.L., Solymos P., Stevens M.H.H., Wagner H. 2013 *vegan: Community Ecology Package. R package version 2.0-10*.

17. Hoeke R., Brainard R., Moffitt R.B., Kenyon J. 2006 Oceanographic conditions implicated in the 2002 Northwestern Hawaiian Islands bleaching event. *Proc 10th Internat Coral Reef Symp*, 718-723.

18. Excoffier L., Foll. M. 2011 Fastsimcoal: a continuous-time coalescent simulator of genomic diversity under arbitrarily complex evolutionary scenarios. *Bioinformatics* **27**, 1332-1334.

19. Yan L.N. and Zhang D.X. 2004. Effects of sample size on various genetic diversity measures in population genetic study with microsatellite DNA markers. *Acta Zool Sinica* ***2***.

20. Kalinowski S.T. 2005. Do polymorphic loci require large sample sizes to estimate genetic distances? *Heredity* **34**, 33-36.

21. Hale M.L., Burg T.M., Steeves T.E. 2012. Sampling for microsatellite-based population genetic studies: 25 to 30 individuals per population is enough to accurately estimate allele frequencies. *PloS one*, **7**, e45170.

22. Karl S.A., Toonen R.J., Grant W.S., Bowen, B. W. 2012. Common misconceptions in molecular ecology: echoes of the modern synthesis. *Mol Ecol* **21**, 4171-4189.

**Table S1.** Colinearity matrix based on Pearson’s r values for all genetic diversity and seascape variables at 13 islands sampled across the archipelago. Column headers correspond to row labels. AR is allelic richness, mtDNA genetic metrics use 47 datasets, nuclear AR uses 11 multi-locus nuclear datasets sampled at 10 islands, GD is gene diversity, F_ST_ is genetic isolation.

|  | 1 | 2 | 3 | 4 | 5 | 6 | 7 | 8 | 9 | 10 | 11 | 12 | 13 | 14 | 15 | 16 | 17 |
| --- | --- | --- | --- | --- | --- | --- | --- | --- | --- | --- | --- | --- | --- | --- | --- | --- | --- |
| 1. Mean mtDNA AR | 1.00 |  |  |  |  |  |  |  |  |  |  |  |  |  |  |  |  |
| 2. Mean NucDNA AR | 0.70 | 1.00 |  |  |  |  |  |  |  |  |  |  |  |  |  |  |  |
| 3. Mean mtDNA GD | 0.94 | 0.54 | 1.00 |  |  |  |  |  |  |  |  |  |  |  |  |  |  |
| 4. Mean mtDNA F_ST_ | -0.67 | -0.39 | -0.60 | 1.00 |  |  |  |  |  |  |  |  |  |  |  |  |  |
| 5. Habitat Area**^†^** | 0.68 | 0.64 | 0.61 | -0.51 | 1.00 |  |  |  |  |  |  |  |  |  |  |  |  |
| 6. Habitat Area**^††^** | 0.72 | 0.76 | 0.56 | -0.36 | 0.80 | 1.00 |  |  |  |  |  |  |  |  |  |  |  |
| 7. Coral Cover | 0.56 | 0.62 | 0.48 | -0.12 | 0.32 | 0.59 | 1.00 |  |  |  |  |  |  |  |  |  |  |
| 8. CCA Cover | 0.52 | 0.09 | 0.66 | -0.02 | 0.41 | 0.31 | 0.39 | 1.00 |  |  |  |  |  |  |  |  |  |
| 9. LGM Habitat Loss | -0.36 | -0.26 | -0.37 | 0.11 | 0.03 | -0.20 | -0.52 | -0.10 | 1.00 |  |  |  |  |  |  |  |  |
| 10. Nearest Neighbor | -0.16 | -0.51 | -0.10 | 0.17 | -0.35 | -0.39 | -0.12 | 0.08 | 0.34 | 1.00 |  |  |  |  |  |  |  |
| 11. Larval Immigration | -0.30 | -0.26 | -0.34 | 0.26 | 0.14 | 0.09 | 0.08 | 0.24 | 0.52 | 0.18 | 1.00 |  |  |  |  |  |  |
| 12. Fish Spp Richness | 0.72 | 0.85 | 0.53 | -0.44 | 0.59 | 0.83 | 0.61 | 0.02 | -0.47 | -0.33 | -0.25 | 1.00 |  |  |  |  |  |
| 13. Coral Spp Richness | 0.53 | 0.23 | 0.33 | -0.52 | 0.57 | 0.59 | 0.52 | 0.11 | -0.18 | 0.39 | 0.31 | 0.63 | 1.00 |  |  |  |  |
| 14. Thermal Stress | -0.62 | -0.59 | -0.54 | 0.17 | -0.44 | -0.66 | -0.42 | -0.20 | 0.48 | 0.24 | 0.48 | -0.69 | -0.16 | 1.00 |  |  |  |
| 15. *M. capitata* AR | -0.47 | -0.29 | -0.43 | 0.05 | -0.33 | -0.53 | -0.47 | -0.31 | 0.41 | 0.20 | -0.08 | -0.59 | -0.39 | 0.09 | 1.00 |  |  |
| 16. Wave Disturbance | -0.10 | -0.26 | -0.01 | -0.35 | -0.18 | -0.49 | -0.73 | -0.28 | 0.33 | 0.21 | -0.40 | -0.35 | -0.27 | 0.37 | 0.25 | 1.00 |  |
| 17. Latitude | -0.01 | -0.21 | 0.08 | -0.40 | -0.13 | -0.43 | -0.71 | -0.26 | 0.23 | 0.15 | -0.52 | -0.26 | -0.28 | 0.22 | 0.26 | 0.98 | 1.00 |

**^†^** Based on bathymetry

**^††^** Based on classified satellite imagery

**Table S2.** Differences in main results based on rarefying allelic richness to 6 haplotypes (RAR 6) versus 10 haplotypes (RAR 10). A. Redundancy analysis; ‘cum.’ indicates cumulative. B. Congruence analysis. C. Seascape model selection analysis. * is p ≤ 0.05, ** is p ≤ 0.01, *** is p ≤ 0.001.

A. Redundancy Analysis

| Dataset | cum. r^2^ | axis 1 r^2^ | axis 2 r^2^ |
| --- | --- | --- | --- |
| RAR 6 | 0.47* | 0.48*** | 0.37*** |
| RAR 10 | 0.49* | 0.54*** | 0.28*** |

B. Congruence

| Dataset | Mean r | t-test | N |
| --- | --- | --- | --- |
| RAR 6 | 0.13 | 2.21* | 36 |
| RAR 10 | 0.16 | 2.38** | 34 |

C. Seascape model selection of composite AR

|  | RAR 6 | | RAR 10 | |
| --- | --- | --- | --- | --- |
| Predictors | Adj. r^2^ | Δ AIC*_c_* | Adj. r^2^ | Δ AIC*_c_* |
| Habitat area, Coral cover | 0.59*** | 0.00 | 0.63* | 0.00 |
| Habitat area, LGM habitat loss | 0.59*** | 0.23 | 0.57* | 0.68 |
| Habitat area, Thermal stress | 0.58** | 0.40 | 0.40 | 6.29 |
| Habitat area | 0.44** | 0.50 | 0.35* | 2.57 |
| CCA cover, Thermal stress | 0.56** | 0.76 | 0.53 | 3.37 |
| Thermal stress | 0.39* | 0.82 | 0.23 | 4.57 |
| % Coral cover | 0.34* | 2.04 | 0.48* | 2.24 |
| % Coral cover, LGM habitat loss | 0.34 | 6.26 | 0.50* | 4.26 |

**Table S3.** Correlation of spatial patterns of allelic richness for 8 fishes with paired nuclear and mitochondrial datasets. All samples rarefied to 10 individuals. Sources indicated by superscripts.

| Species | Second Marker type | Loci | Pearson’s r value | N islands |
| --- | --- | --- | --- | --- |
| *Abudefduf abdominalis*^1^ | Nuclear sequence | 3 | 0.83 | 8 |
| *Abudefduf vaigiensis*^1^ | Nuclear sequence | 3 | 0.61 | 6 |
| *Epinephelus/Hyporthodus quernus*^2^ | Microsatellite | 6 | 0.32 | 9 |
| *Etelis coruscans*^3^ | Microsatellite | 8 | 0.13 | 7 |
| *Etelis marshi*^3^ | Microsatellite | 9 | 0.06 | 9 |
| *Lutjanus kasmira*^4^ | Nuclear sequence | 3 | 0.26 | 8 |
| *Pristipomoides filamentosus*^5^ | Microsatellite | 13 | 0.11 | 8 |
| *Zebrasoma flavescens*^6^ | Microsatellite | 13 | 0.57 | 12 |

**Source Data**

1. Coleman RR, Gaither MR, Kimokeo B, Stanton FG, Bowen B, Toonen RJ (2014) Large-scale introduction of the Indo-Pacific damselfish Abudefduf vaigiensis into Hawai'i promotes genetic swamping of the endemic congener A. abdominalis. *Molecular Ecology*, 23, 5552-5565.

2. Rivera MAJ, Andrews KR, Kobayashi DR, Wren JLK, Kelley C, Roderick GK, Toonen RJ (2011) Genetic analyses and simulations of larval dispersal reveal distinct populations and directional connectivity across the range of the Hawaiian Grouper (*Epinephelus quernus*). Journal of Marine Biology, 2011, 1–11.

3. Andrews KR, Moriwake VN, Wilcox C, Grau EG, Kelley C, Pyle RL, Bowen BW (2014) Phylogeographic analyses of submesophotic snappers *Etelis coruscans* and *Etelis "marshi"* (Family Lutjanidae) reveal concordant genetic structure across the Hawaiian Archipelago. Plos One 9, e91665-e91665.

4. Gaither MR, Bowen BW, Toonen RJ, Planes S, Messmer V, Earle J, Robertson DR (2010) Genetic consequences of introducing allopatric lineages of Bluestriped Snapper (*Lutjanus kasmira*) to Hawaii. Molecular Ecology, 19, 1107-1121.

5. Gaither MR, Jones S a, Kelley C, Newman SJ, Sorenson L, Bowen BW (2011) High connectivity in the deepwater snapper *Pristipomoides filamentosus* (Lutjanidae) across the Indo-Pacific with isolation of the Hawaiian archipelago. PloS one, 6, e28913.

6. Eble J, Toonen R, Sorenson L, Basch L, Papastamatiou Y, Bowen B (2011) Escaping paradise: larval export from Hawaii in an Indo-Pacific reef fish, the yellow tang *Zebrasoma flavescens*. Marine Ecology Progress Series, 428, 245–258.

**Figure S1. Principal Components Analysis of seascape factors at all islands.** See Table 2 in main text for explanation of factors.


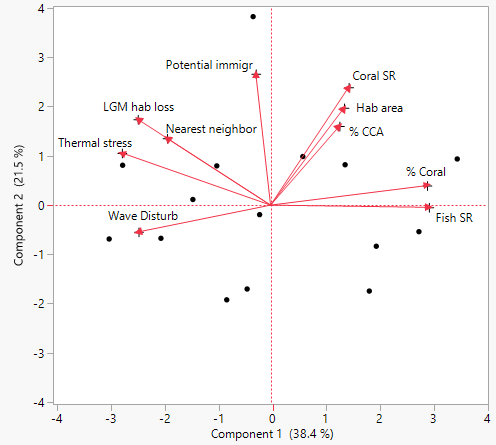


*Oahu*

*Hawa*

*Maui*

*Lana*

*Molo*

*Midw*

*Kure*

*Niho*

*Lays*

*Pear*

*Niih*

*Lisi*

*Fren*

*Maro*

*Kaua*

**Figure S2. Congruence of spatial patterns of allelic richness across species.**  Distribution of correlations between individual species’ mtDNA allelic richness and the composite mtDNA mean for 34 species sampled at ≥ 5 islands. Box plot horizontal lines indicate maxima and quartiles, diamond is centered on mean with tips at 95% confidence interval; red bracket shows range of 50% observation density.

**
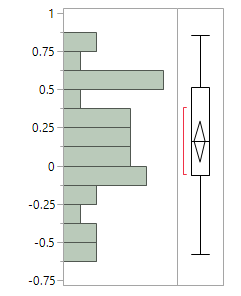
**

**Figure S3**. **Plots of composite mtDNA allelic richness against 12 factors.** Numbers indicate islands in order from southeast to northwest (1. Hawai‘i, 2. Maui, 3. Moloka‘i, 4. Oahu, 5. Kaua‘i, 6. Ni‘ihau, 7. Nihoa, 8. French Frigate Shoals, 9. Maro, 10. Laysan, 11. Pearl and Hermes, 12. Midway, 13. Kure).

**
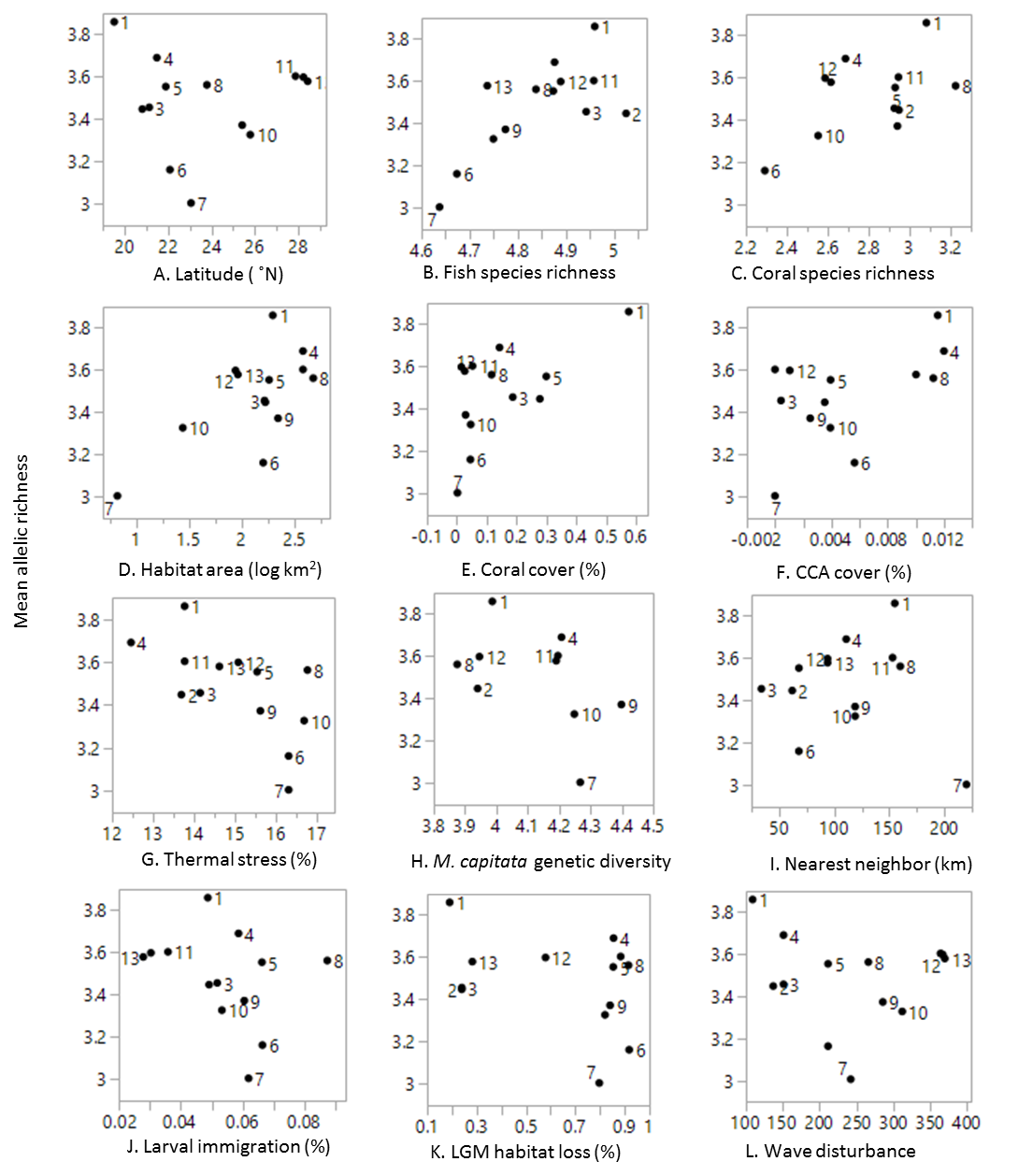
**

**Fig. S4.** **Output of genetic simulations.** Plots show allelic richness along a linear habitat array with migration among neighboring populations only. Solid squares correspond to the scenario where all populations have equal sizes (N_e_ = 1000); circles correspond to a scenario were the two marginal populations are three times larger than the others (N_e_ = 3000 vs N_e_ = 1000).

**Figure S5. Redundancy analysis relating genetic diversity, seascape variables and species’ traits.** Predictive power of species traits (blue font) for the strength of spatial correlation of each species’ AR values with seascape factors (red font). Seascape factors adjacent to trait vectors are under positive influence, factors opposing are under negative influence; these associations are driven by the species names clustered adjacent or opposing to the vectors (black font, see Dataset S1 for full names).

**
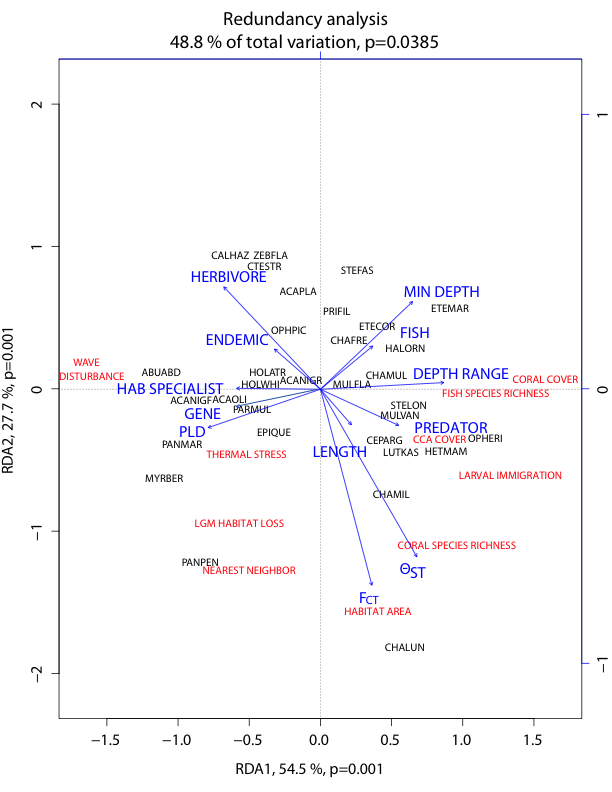
**

**Figure S6. Modeled larval immigration rates.** A. Map of Hawaii overlaying estimates of ‘larval immigration’ (i.e., in-closeness centrality; green circles) and pairwise rates of larval exchange (greyscale lines), with southeastward movement above the archipelago and northwestern movement below the archipelago. Island to the south is Johnston Atoll. B. Plot of Larval Immigration against latitude for the 13 Hawaiian Islands analyzed.

**
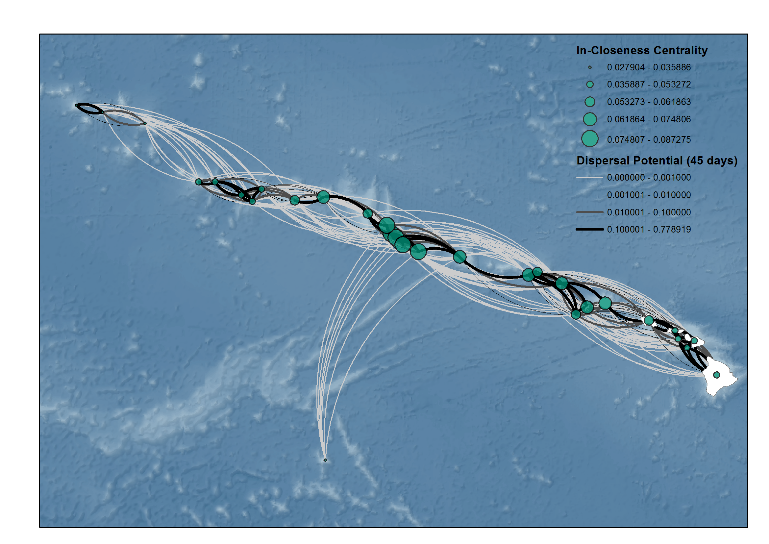
**

**A**

**B**

**
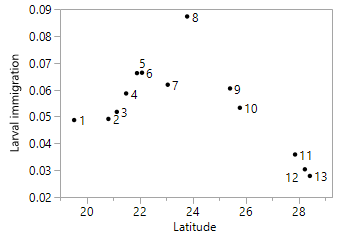
**

**Fig. S7.** **The distribution of species’ correlations of mtDNA AR to habitat area.** Solid arrow indicates the composite AR correlation to habitat area inclusive of all species (r = 0.66). Shaded units indicate 8 species with highest individual correlations. Dashed arrow indicates the composite AR correlation to habitat area with these 8 species omitted from the mean (r = 0.61). Box plot horizontal lines indicate maxima and quartiles, diamond is centered on mean with tips at 95% confidence interval; red bracket shows range of 50% observation density; A: for all species (mean r = 0.05, N = 39), B: when 8 species are omitted (mean r = -0.10, N = 31). Identities of omitted species are: *C. exarata, C. lunulatus, C. miliaris, H. ornatissimus, L. kasmira, M. vanicolensis, O. erinaceus and P. pencillatus*; three are invertebrates and three are endemics.


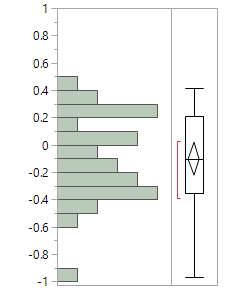

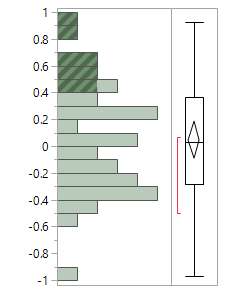


A

B

**Fig S8. Comparison of correlations of mean allelic richness to fish species richness**. A. Mean mtDNA AR based on all species; B. Mean mtDNA AR on all 37 fishes. Red lines include all islands; green lines exclude islands numbered 2 (Maui) and 4 (Molokai) which are outliers in plot B.

A

B


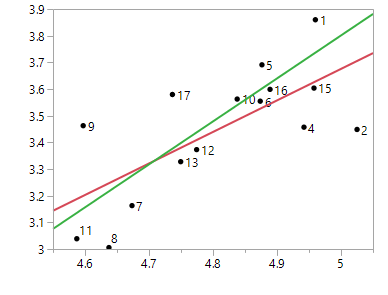

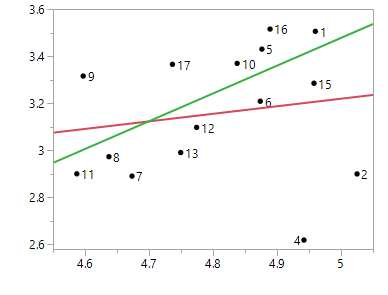


Natural Log of Fish Species Richness
